# Predicting developmental outcomes in middle childhood from early life language and parenting experiences

**DOI:** 10.1111/bjdp.12427

**Published:** 2022-07-26

**Authors:** Sophie von Stumm, Jelena O'Reilly, Katrina d'Apice

**Affiliations:** ^1^ Department of Education University of York York UK; ^2^ Bristol Medical School University of Bristol Bristol UK

**Keywords:** academic performance, language, naturalistic observation, parenting, strengths and difficulties

## Abstract

Children's early life experiences of language and parenting are thought to have pervasive, long‐term influence on their cognitive and behavioural development. However, studies are scarce that collected naturalistic observations to broadly assess children's early life experiences and test their associations with developmental outcomes in middle childhood. Here, we used digital audio‐recorders to collect three full days of naturalistic observations from 107 British families with children (46 boys) aged 2–4 years, of whom 89 participated in a follow‐up assessment four years later when the children were 5–8 years old. We found that children's early life experiences of language and parenting were not significantly associated with their later language ability, academic performance and behavioural outcomes. We explore differences in methodology, sample characteristics and the role of developmental periods as possible explanations for the discrepancy in findings between the current and previous studies.

**Statement of Contribution**

***What is already known on this subject?***

- Early life experiences of language and parenting influence children's development
- Previous research focused on either language or parenting but rarely studied both
- Naturalistic observation studies of children's early life experiences are scarce

***What does this study add?***

- Using digital audio‐recorders, three days of naturalistic observations were collected
- 107 British families participated when children were 2–4 years old and followed up four years later
- Early life experiences of language and parenting were not significantly associated with children's later development

## BACKGROUND

The family environments that children experience in their early life have pervasive, long‐term influence on their subsequent development. Two pivotal characteristics of the family environment are the language (Hart & Risley, 1995) and the parenting (Stein et al., 2013) that children are exposed to. Both have been shown to influence children's development: the quantity and quality of language that children experience contributes to their cognitive and verbal development (Bornstein, 2002; Greenwood et al., 2011; Rowe, 2012; Zimmerman et al., 2009), while parenting shapes children’ social–emotional competencies. Specifically, positive parenting, which refers to the warmth, encouragement and engagement that parents regard their children with (Bennetts et al., 2016), improves children's behavioural outcomes (Stein et al., 2013), while critical parenting, characterized by negative attitudes and disapproval, predicts externalizing and internalizing behaviour problems in children (Eisenberg et al., 2005; Gulenc et al., 2018; Pinquart, 2017; Sher‐Censor et al., 2018). The influence of language experiences on children's cognitive and verbal development is typically of medium effect size across studies (d'Apice et al., 2019; Wang et al., 2020); by comparison, associations between parenting and child behavioural outcomes tend to be weaker (Pinquart, 2017).

Although a plethora of earlier studies investigated the role of early life experiences for child development, more research is needed for three reasons. First, most prior studies focused on one kind of early life experience, for example the number of words that a child is exposed to and its influence on one kind of developmental outcome, such as a child's attained vocabulary (Rowe, 2012; Wang et al., 2020). This approach ignores that parenting behaviours and language exposure are intertwined and together with many other factors, exert influence on development, possibly across domains. This point was illustrated by one previous study that assessed parenting (i.e., maternal sensitivity) and language exposure (i.e., mothers' stimulation of child language use) in 146 mother–child dyads, who were observed during 10‐minute play activities at the ages 14, 24 and 36 months (Vallotton et al., 2017). Maternal sensitivity and language stimulation correlated on average .50 across assessment ages, with both language stimulation and sensitivity being independently and differently associated with children's vocabulary growth throughout toddlerhood (Vallotton et al., 2017). These findings suggest that (a) parenting and language exposure are related aspects of early life environments and (b) that charting the interplay between both is key for improving our understanding of child development.

Second, previous studies in this area often collected naturalistic home observations of early life environments from small samples for relatively short periods of time that ranged from 10 minutes to 12 hours (d'Apice et al., 2019; Purpura, 2019; Wang et al., 2020), although there are some notable exceptions (Gilkerson et al., 2018; Griffin & Morrison, 1997; Vallotton et al., 2017). Naturalistic observations have greater ecological validity than lab‐based studies and are free from biases that typically affect self‐reported measures, yet they have been expensive to collect (Hart & Risley, 1995). Recently, technical innovations for research observation tools, in particular digital audio‐recorders (Christakis et al., 2009; Gilkerson et al., 2018), have lowered the costs for collecting naturalistic observations from families. These new tools make it now possible to add to the existing body of empirical evidence by assessing larger family samples for longer observation periods.

Third, most previous studies in this area have focused on development during toddlerhood and in preschool children (Briggs‐Gowan et al., 2006; Durham et al., 2007; Sher‐Censor et al., 2018) but less is known about the influence of early life experiences for developmental outcomes in middle childhood or during primary school (Bleses et al., 2016; Lee, 2011). To expand our knowledge of this period of child development, we present here findings from a longitudinal study that tested the association of early life language and parenting experiences, which were extracted from sustained naturalistic observations, with key developmental outcomes in middle childhood, including language ability, academic performance and behavioural outcomes.

### Early life language experiences and developmental outcomes

Language development, particularly in the early stages of life, is influenced by children's inherent propensity for language ability and the language environments that they are exposed to (i.e., proximal processes; Bronfenbrenner & Ceci, 1994). The language environment can be characterized by the quantity of adult speech – the number of words that children hear – and the quality of adult speech, for example the lexical and vocabulary sophistication that children are exposed to (Ochs & Schieffelin, 2011). The quantity of adult speech has been shown to be associated with children's language skills throughout toddlerhood and the preschool years. For example in 50 parent–child dyads, the total number of words that children aged 18 months heard over the course of 90 min correlated .30 and above with their vocabulary up to three years later (Rowe, 2012). Likewise, the quality of adult speech is positively associated with children's language development: Children of mothers, who used more varied vocabulary during video‐taped interactions with their child, showed faster growth in vocabulary between 14 and 36 months (Pan et al., 2005).

Although children's language environment seems to affect their early language development, the pervasiveness of this influence over time is unclear, because few studies exist that included follow‐up assessments in middle childhood. In addition, the available research has produced inconsistent findings. For example, some studies suggested that the influence of the family language environment weakens once children start school (Broberg et al., 1997; Melhuish et al., 2008), while other studies find evidence for its continuous impact (Griffin & Morrison, 1997). In a large sample of 2354 children, the effect of the family language environment, including joint‐book reading and teaching letters and rhymes, on children's language development and school performance decreased from age 3 through 7 years (Melhuish et al., 2008). Factors other than the family language environment, such as schooling and peers, appear to gain in influence on children's language and academic development as they grow older. Evidence from a smaller sample aligns with this conclusion: in 146 Swedish children, early life home environment predicted verbal ability when children were aged 2 and 4 years but when they were 8 years old, no association was observed (Broberg et al., 1997). Yet, another longitudinal study of 295 children revealed that literacy environments during kindergarten age correlated .55 with receptive vocabulary and .49 with reading skills at 8 years of age (Griffin & Morrison, 1997).

Early life language environments are not only associated with children's language abilities but also with their cognitive development and academic performance (Duncan et al., 2007; Durham et al., 2007; Gilkerson et al., 2018; Peng et al., 2019; Peng & Kievit, 2020; von Stumm et al., 2020). Some argue that the intersection of cognitive, language and academic abilities stems from shared genetic and environmental influences that affect development across domains (Dickens & Flynn, 2001; Peng & Kievit, 2020). Others contend that cognitive and academic abilities become related to one another over the course of development because of mutually beneficial interactions that occur between originally uncorrelated cognitive processes (Van Der Maas et al., 2006). Notwithstanding these different perspectives, we would expect that early life language experiences benefit the development of a broad nexus of cognitive abilities that are interrelated, rather than being specific to one (Peng et al., 2019).

### Early life parenting experiences and development

Children's behavioural outcomes are often conceptualized as strengths and difficulties that manifest themselves in conduct, emotions, hyperactivity, peer relationships and prosocial behaviour (Goodman, 1997). Children's behavioural difficulties can be differentiated into internalizing problems, for example anxiety, withdrawal and inhibition, and externalizing problems, such as aggression, hyperactivity and impulsivity (Briggs‐Gowan et al., 2006). The development of externalizing and internalizing problems is thought to be influenced by the parenting that the children experience in early life (Aunola & Nurmi, 2005). Overall, positive parenting is associated with fewer externalizing and internalizing behaviours, while critical parenting is associated with an increase in these behaviours (Akcinar & Baydar, 2016; Boeldt et al., 2012; Denham et al., 2000). For example, children who experience supportive parenting with clear instructions and boundaries suffer fewer externalizing behaviour problems throughout middle childhood, while children who face higher levels of parental anger show more externalizing problems (Denham et al., 2000). These findings were consistent across different assessment methods (i.e., lab‐based observations and self‐reports; Denham et al., 2000). They were also corroborated by a longitudinal study of 547 children, for whom warm and encouraging parenting during infancy and toddlerhood was associated with reduced externalizing behaviour problems between the ages of 7 and 12 years (Boeldt et al., 2012). Conversely, in a 4‐year longitudinal study of 1009 mother–child dyads, physically harsh parenting led to increased externalized behaviour in children across observation‐based assessments and maternal reports (Akcinar & Baydar, 2016). In summary, previous research has frequently reported meaningful associations between parenting and children's behavioural outcomes.

Some studies have suggested that parenting may also influence children's academic ability, in addition to their behavioural outcomes. For example in 55 mother–child dyads, mothers' positive parenting, as indexed by their smiles and displays of positive emotions towards their children, predicted the children's kindergarten competency, accounting for 30% of the variance (Pianta et al., 1997). In a study of 304 8‐year olds, mothers' responsiveness towards their child was associated with higher academic performance, while controlling and punitive parenting strategies were linked with poor academic outcomes (Chen et al., 1997). Furthermore in a sample of 66 children, parenting characterized by reasoning, encouragement and overall respect for the child's autonomy benefitted school grades between the ages of 8 and 11 years (Grolnick & Ryan, 1989). Thus, early life experiences of parenting are likely to influence development across domains, including behavioural and academic outcomes.

### The current study

With the current study (preregistration: https://osf.io/y9tn4/), we aimed to investigate the association of early life language and parenting experiences with cognitive and language ability, behavioural outcomes and academic performance in middle childhood. Using digital audio‐recorders (i.e., LENA™; LENA Research Foundation, 2009), we unobtrusively observed 107 families from Britain who had a child aged 2–4 years for three full days (d'Apice et al., 2019). Children wore the audio‐recorders in custom‐made t‐shirts; everything that the child heard or said was recorded within a 6‐foot radius for up to 16 hours per day. We used these recordings to estimate the quantity and quality of the language and the parenting that children experienced in their respective family homes. Four years later, we followed up the families and assessed children's cognitive and language ability, behavioural outcomes and academic performance.

Based on the previous literature, we hypothesized that children's early life experiences of language and parenting would predict their cognitive and language ability, behavioural outcomes and academic performance in middle childhood, after controlling for their earlier cognitive, language and behavioural outcomes, as well as for family characteristics (i.e., confounders). We thought that early life language experiences would predict cognitive and language abilities, as well as academic achievement in middle childhood. Conversely, we hypothesized that early life experiences of parenting would primarily predict children's later behavioural adjustment. We also expected that children's earlier outcomes would be associated with their later outcomes within the respective developmental domains; for example, language ability in early childhood would be positively associated with language ability in middle childhood, and behavioural outcomes in early life would relate to behavioural outcomes in middle childhood.

## METHOD

### Sample and procedures

Between November 2014 and August 2016, we recruited 107 families from Southeast London through nurseries, Facebook and research contacts for our first wave of data collection (herein T1), of whom 89 (83% retention rate) completed a follow‐up survey that was circulated in June and July 2019 (herein T2). Ethics approval for data collection at T1 titled ‘Cognitive Development and Linguistic Home Environment’ was obtained from Goldsmiths University of London in September 2014; ethics approval for data collection at T2 titled ‘LENA 2.0: Following up a naturalistic home observation study’ was obtained from the University of York in May 2019.

The 89 families who completed T1 and T2 included 88 mothers, who were on average aged 37 years (*SD* = 4.21) at T1, 64 fathers aged 39 years (*SD* = 4.82) at T1, as well as 89 children (46 boys), who were aged 2.6 years (*SD* = 0.54, range = 1.79–3.93) at T1 and 6 years (*SD* = 0.68, range = 5.25–8.00) at T2. The difference in age range at T1 and T2 is because the recruitment and data collection period at T1 took more than one year (i.e., from November 2014 and August 2016), with some of the older children participating earlier in the study and some younger ones later, while data at T2 were collected within 1 month. Demographics were assessed at T1: parents had spent on average 33.42 years in the United Kingdom (*SD =* 10.67, range = 0–53 years), with the vast majority being born in Britain and native speakers of English (99%). Most parents in the sample held university degrees (85% of mothers and 80% of fathers), were married co‐parents (98%), and had been living together for 4 or more years (92%). Although families varied in socio‐demographic background, they were on average of high SES and are only representative of some British parents.

Almost all children attended primary school at T2 (96%), except for three who were home schooled and one who was due to start school abroad. About half (58%) of the children had siblings who lived in the same household.

A logistic regression model showed that the sample at T2 did not differ from the sample at T1 in the variables assessed at T1 (i.e., SES, child behaviour, cognitive ability and language; home language input, including number of words and lexical diversity; critical and positive parenting; child's age at T1 and child's gender; *p* > .05 in all cases).

### Measures

#### Time 1 measures

##### Home language input

LENA™ digital language processers (DLPs) are small lightweight audio‐recorders that can be inserted into specially designed child clothing. The DLPs were delivered to the participating families' homes after they had registered for the study and completed an online survey. Parents were instructed to conduct day‐long recordings on three days when their child was not attending nursery or any other formal childcare setting. The DLPs recorded all sounds within a 6‐foot radius for up to 16 hours per day. *Adult word counts* were used as the measure of the quantity of language input (i.e., the total number of words spoken by adults), which were extracted using the LENA™ software. LENA word count estimates were successfully validated against a subsample of human‐transcribed excerpts with word counts computed using Computerized Language Analysis software (CLAN; MacWhinney, 2000). LENA‐ and CLAN‐based adult word counts across 64 transcribed 5‐minute excerpts were strongly positively correlated (*r* = .79 and *r* = .83 once adjusted for recording distance, *p* < .001; d'Apice et al., 2019). *Adult lexical diversity* was used as the measure of the quality of home language input. Lexical diversity was indexed by D‐scores (McKee et al., 2000), which were computed using CLAN for each family from six transcribed 5‐minute excerpts (i.e., 2 per day for 3 days, so 6 excerpts with a total of 30 min per family). For transcription, we selected per family the two 5‐minute recording excerpts per day that registered the highest number of conversational turns in LENA™ (i.e., total number of conversational interactions that a study child had with an adult, in which one speaker initiates and the other responds within 5 s), one from between 8 a.m. and 11 a.m. and one from between 5 p.m. and 8 p.m. These excerpts offer the richest linguistic and behavioural data for our analyses (see d'Apice et al., 2019, for more details).

##### Parenting

Parenting was rated by two trained research assistants using the transcribed 5‐minute excerpts (i.e., 6 excerpts per family of overall 30 min recordings). Two research assistants were trained by two of the authors to rate parenting using eight items selected from the Parenting Styles and Dimensions Questionnaire (PSDQ, Robinson et al., 1995) and two items from ‘opportunities for variety in daily stimulation’ subscale of The Home Observation for Measurement of the Environment (HOME, Caldwell & Bardley, 1984). The frequency at which each of the 10 behaviours occurred during the 5‐minute excerpts was rated on a 5‐point scale (e.g., 1 = *never*, 5 = *always*). The recordings were randomized to avoid ratters listening to excerpts from the same family in succession.

To assess the validity of our parenting ratings, we first tested the two research assistants' inter‐rater agreement for parenting ratings in a subsample of 51 families for whom there were two (or more) recordings of the same parent and child; for those families with 2+ recordings, two recordings were randomly selected. Inter‐rater agreement was on average 73%. We then tested the inter‐rater agreement for parenting ratings across all recordings (i.e., including recordings with additional parents and children present), which was also 73%. Because absolute within‐family differences in parenting across recordings were small, we computed overall parenting behaviour scores across both parents per family. These summary scores were then subjected to factor analysis, from which two factors emerged, each with three items loading above .35: ‘positive parenting’ (e.g., responsive, expression, novelty) and ‘critical parenting’ (e.g., threatening, criticizing, shouting). Composite scores were created by summing the corresponding factor's items, with higher scores representing more positive and critical parenting behaviours, respectively. Further details on the parenting measure are reported by d'Apice et al., (2019).

##### Child language

Children's lexical diversity was indexed by D‐scores (as described above for adults), which was based on children's language from the transcribed 30‐minute excerpts from the LENA recordings.

##### Child cognitive ability

The Parent Report of Children's Abilities (PARCA; Oliver et al., 2002; Saudino et al., 1998) was used to index children's cognitive skills. Items were selected from the PARCA versions for children aged 2 and 3 years, in line with the age range of the current sample. Parents completed a testing booklet together with their child at home, including three sections on drawing, copying and matching. Composite scores for the three sections (i.e., drawing, copying and matching) correlated .33, .42 and .51. A single standardized score was created by summing and *z*‐transforming composite scores for the three sections of the PARCA testing booklet.

##### Child behaviour

Child behaviour was rated by two trained research assistants using the 5‐minute audio excerpts per family that were also used to rate parenting and to extract children's and parents' D‐scores (see above). Two research assistants were trained to rate the child behaviour from the audio‐recordings according to 10 adjectives describing internalizing, externalizing and hyperactive behaviours, using a scale from 1 to 10. Adjectives were generated based on the Child Behaviour Checklist for Ages 1.5–5 years (CBCL; Achenbach & Rescorla, 2000) and the Rutter Scale (Rutter et al., 1970). A rating of 5 indicated ‘normal behaviour’ while deviations from 5 indicated atypical behaviour (i.e., 1 = notably absent, 5 = normal, 10 = excessive). For example, if a child cried during the recording in response to a negative event (e.g., being hit by a sibling), the behaviour would be rated as ‘normal’ with 5; if a child cried throughout the recording for no clear reason, the child would receive a rating higher than 5 for being tearful. The inter‐rater agreement was 79% on average, and factor analysis suggested two factors: ‘internalizing behaviours’ with four items loading above .35 (e.g., anxious, worried, tearful, depressed) and ‘externalizing behaviours’ with six items loading above .35 (e.g., restless, impatient, distracted, aggressive, irritable, disobedient). Composite scores were created by summing the items for each factor.

##### Family factors

Socio‐economic status (SES) was based on a composite of three *z*‐transformed measures: (i) parents' highest level of education; (ii) the MacArthur Scale of Subjective Social Status (Adler et al., 2000), whereby parents indicated on a ladder drawing, whose lowest rung represents the people with the least resources and power in society and the highest rung those with most, their perceived standing in society; and (iii) an overcrowding index (i.e., total number of rooms/total number of adults and children in the household). Data on educational status and for the MacArthur Scale was available from both parents for 63 families. For all other families, education and MacArthur scale were available from one parent. Data were also collected on birth order and child age and gender.

#### Time 2 measures

##### Child language ability

Child language ability was measured by a parent report based on the Developmental Vocabulary Assessment for Parents (DVAP; Libertus et al., 2015), whose items are adapted from the Peabody Picture Vocabulary Test (PPVT; Dunn & Dunn, 2007). Parents were asked to indicate which words they had heard their child produce from a list of 60 lexical items of increasing difficulty. Responses to the items were coded as false (0) or correct (1) (i.e., heard vs. not heard, respectively).

##### Child academic performance

Three measures assessed children's academic performance. First, parents were asked to rate how well they thought their child performed in school relative to the other children in the same school class using a slider scale from 0 to 100, with a score of 0 indicating the child performed worst and a score of 100 the child performed best. Second, our survey asked parents to report children's scores on two primary school assessments that are mandatory for children to complete in England (i.e., the Phonics Screening Check and Key Stage 1 Standard Attainment Tests; SATs), but too few parents (*N* = 15) provided this information to be used in our analyses. Finally, a 6‐item parent questionnaire was developed for this study to capture children's attitudes towards learning in school. Items were as follows: ‘does your child enjoy attending school?’, ‘does your child enjoy doing homework?’, ‘is your child excited by the work in school?’, ‘does your child care about doing well in school?’, ‘does your child get on with the class teacher?’ and ‘does your child make good learning progress in school?’. Items were rated as ‘not at all’ (0), ‘a moderate amount’ (1) or ‘a great deal’ (2). Scores were summed for each child, with a higher score indicating more positive school attitudes.

##### Child behaviour

Child behaviour was measured by parent report, using the Strengths and Difficulties Questionnaire (SDQ), which has been validated for completion by parents for children aged 2–17 years (Goodman, 1997). The SDQ comprises 25 items to assess five domains: hyperactivity (e.g., ‘cannot sit still for long’), emotional symptoms (e.g., ‘often seems worried’), conduct problems (e.g., ‘often fights with other children or bullies them’), peer problems (e.g., ‘tends to play alone’) and prosocial behaviour (e.g., ‘shares readily with other children’). Each item is scored on 3‐point scale (i.e., ‘not true’, ‘somewhat true’ and ‘certainly true’). Scores from the hyperactivity, emotional symptoms, conduct problems and peer problems scales were summed to create a Total Difficulties Score (theoretical range: 0–40, with higher scores indicating greater difficulties). Scores from the Prosocial Scale were summed to provide a prosocial behaviour score (theoretical range 0–10, with higher scores indicating more prosocial behaviour).

### Statistical approach

Our analyses were preregistered (preregistration: https://osf.io/y9tn4/). To test the study's main hypotheses, a series of multiple linear regression models predicted each of the T2 child outcomes from the T1 measures of early life experiences, children's earlier developmental outcomes and confounding family characteristics. In a first step (Model 1), confounding family characteristics (i.e., birth order and SES) and T1 early life experiences (i.e., adult word counts, lexical diversity and positive and critical parenting) were entered, to estimate the relation between children's early life experiences and their outcomes in middle childhood. In the second step (Model 2), T1 child outcomes (i.e., lexical diversity, PARCA scores and internalizing and externalizing behaviours) were added to assess whether early life experiences continued to account for variance in children's later outcomes after controlling for their earlier characteristics. We adjusted all child measures for gender and their respective ages at T1 and T2, retaining unstandardized regression residuals for all further analyses.

We examined *p‐*values to assess whether individual predictors were significant (*p* < .05), along with the *R* ^2^ statistic to assess whether adding T1 child outcomes to the model accounted for more variance in child outcomes at T2. Including all T1 early life experience predictors in Model 1 and all T1 child outcomes in Model 2 enabled testing for domain‐specific relations between early life experiences and later outcomes, while also controlling for earlier child outcomes. If, for example, early language exposure predicted children's later vocabulary but not their behaviour, then we can conclude that language exposure has a domain‐specific rather than a general association with child development. Data were missing due to loss at follow‐up from T1 to T2 and because not all families completed all measures (Table 1). Data were missing at random; accordingly, we report correlations after pairwise omission (Table 2) and regression results after listwise omission (Table 3).

**TABLE 1:** Descriptive statistics for all study variables

Variable	*N*	Mean	*SD*	Min.	Max.	Skew	Kurtosis	α
Adult word counts	107	54,065.00	17,135.33	15,038.02	99,684.85	0.37	0.05	–
Adult lexical diversity	107	108.45	14.64	32.89	133.8	−1.54	5.29	–
Positive parenting	104	2.78	0.54	1.04	4.14	−0.52	0.83	.71
Critical parenting	104	1.01	0.04	1.00	1.33	4.52	24.88	.53
Child lexical diversity	107	63.98	18.91	25.46	115.29	0.00	−0.33	.68
PARCA–Standardized	104	0.00	1.00	−2.35	2.54	−0.17	−0.31	–
Internalizing behaviour	107	4.97	0.11	4.71	5.26	0.07	−0.38	.70
Externalizing behaviour	107	5.09	0.08	4.89	5.25	−0.09	−0.55	.63
DVAP	89	56.61	6.17	19.07	61.07	−2.91	13.34	.97
Academic performance	85	76.25	17.05	18.03	107.84	−0.82	0.87	–
Attitudes towards school	83	9.98	1.77	4.27	13.08	−0.91	0.58	.74
Total Difficulties Score	89	6.86	4.11	−0.29	21.26	0.89	1.02	.58
Prosocial Behaviour Score	89	8.15	1.82	2.40	10.67	−0.74	−0.11	.73
SES index	107	−0.02	0.56	−1.69	0.95	−0.54	0.01	.78

*Note*: Child measures have been corrected for child's age and gender at T1 and T2. Parenting data and PARCA scores at T1 was only available for 104 families; academic performance was only available for 85 children, because 4 did not attend school at T2; likewise, attitudes towards school was only available for *N* = 83, because 4 children did not attend school at T2 and two parents did not complete the items. Adult word counts refer to total words spoken by adults within 6‐foot radius of study child over 3‐day period.

Abbreviations: DVAP, Developmental Vocabulary Assessment for Parents; PARCA, Parent Report of Children's Abilities; SES, socio‐economic status.

**TABLE 2:** Correlations after pairwise omission

	1	2	3	4	5	6	7	8	9	10	11	12	13
1. Adult word counts	–												
2. Adult lexical diversity	.09	–											
3. Positive parenting	.31**	−.09	–										
4. Critical parenting	−.07	.12	−.19	–									
5. Child lexical diversity	.14	.42	.12	−.06	–								
6. PARCA	.39**	.14	.20*	.10	.26**	–							
7. Internalizing behaviour	−.12	−.14	−.06	.29**	−.17	.05	–						
8. Externalizing behaviour	−.06	.16	−.30**	.22*	−.01	.20	.27**	–					
9. DVAP	.07	.09	.02	.02	.23*	.20	.06	−.07	–				
10. Academic performance	.00	−.03	−.09	.10	.03	.41**	.11	.03	.32**	–			
11. Attitudes towards school	−.1	−.14	−.12	−.04	−.01	.18	.14	.01	.15	.38**	–		
12. Total Difficulties Score	.14	.10	.23*	.05	.15	.06	−.07	.07	−.20	−.21	−.38**	–	
13. Prosocial Behaviour Score	.06	−.13	.00	.10	.07	.06	.16	−.03	.33**	.09	.25*	−.45**	–
14. SES index	.12	.22	.06	−.14	.30**	.10	−.07	−.04	.04	−.06	−.06	−.02	.09

*Note*: Child outcomes adjusted for age and gender. Word counts adjusted for recoding duration. Lexical diversity and parenting adjusted for number of available recordings.

Abbreviations: DVAP, Developmental Vocabulary Assessment for Parents; PARCA, Parent Report of Children's Abilities; SES, socio‐economic status.

* <.05

** <.01.

**TABLE 3:** Model fitting results predicting T2 cognitive, language and behavioural child outcomes from T1 early life experience measures, child outcomes and controls

	DVAP		Academic performance		Attitudes towards school		Total difficulties score (SDQ)		Prosocial behaviour score (SDQ)	
	Model 1	Model 2	Model 1	Model 2	Model 1	Model 2	Model 1	Model 2	Model 1	Model 2
*R* ^2^	−.04	.01	−.05	.10	−.02	.01	.05	.05	.03	.03
*F*‐statistic	0.35 (*df =* 6;79)	1.06 (*df =* 10;72)	0.29 (*df =* 6;75)	1.91 (*df =* 10;69)	0.73 (*df =* ‑6; 73)	1.07 (*df =* 10, 67)	1.82 (*df =* 6;79)	1.45 (*df =* 10;72)	1.44 (*df =* 6;79)	1.33 (*df =* 10;72)

*Note*: Degrees of freedom vary across models due to missing data; respective models were fitted to families with complete data. In Model 1, T1 measures were entered as predictors; in Model 2, T1 and T2 measures were entered as predictors.

## RESULTS

Descriptive statistics for all study variables are shown in Table 1, after adjusting for children's gender and age at T1 and T2, respectively. Our scale measures showed good internal consistency. Out of our 14 variables, three had non‐normal distributions with skew and kurtosis values exceeding ±1.5.

### Stability of child outcomes over time

Table 2 shows the correlations of all T1 and T2 measures. PARCA scores at T1 were significantly and positively correlated with the parent‐reported measure of child language (i.e., DVAP) and academic performance at T2. Child lexical diversity at T1 was significantly associated with child language at T2, but not with academic performance. Neither PARCA scores nor child lexical diversity at T1 were significantly related to attitudes towards school at T2. These findings suggest some stability of traits related to cognitive ability over time.

For the behavioural outcomes, externalizing and internalizing behaviours at T1, which were derived from researcher ratings of audio‐recordings, were not associated with parent‐rated SDQ scores at T2, suggesting that child behaviour was not stable across time and assessment methods in our sample.

### Associations between early life experiences and middle childhood outcomes

Of the 10 multiple regression models that we tested, none were associated with a significant *F*‐statistic (Table 3), suggesting that the models were poor representations of our data. We report the full regression model results with coefficients in our Supplementary Materials. Because the overall models were not significant, we refrain from interpreting individual coefficients that were associated with *p* < .05.

## DISCUSSION

Our study sought to test the predictive validity of children's early life experiences of language and parenting for their later development, using naturalistic observations over three days in a sample of British families. Children's early life experiences were poor predictors of their language, behaviour and academic outcomes in middle childhood in our sample, because none of the fitted regression models were significant. Although these findings may seem at odds with some earlier reports (Gilkerson et al., 2018; Griffin & Morrison, 1997; Hart & Risley, 1995; Hoff, 2006), they align with a recent study that employed similar methods as we did (Merz et al., 2020). Specifically, Merz et al., (2020) found no significant association between children's language experience, including the quantity of adult words and the number of conversational turns that they experienced aged 5–9 years and their reading ability in a sample of 76 families from New York.

Notwithstanding the poor fit of the regression models, our study yielded several noteworthy findings. First, our results suggest that children's language showed some stability across time, as lexical diversity at age 2–4 years was positively correlated with children's vocabulary four years later. This finding aligns with previous reports of positive associations between early child vocabulary and later language performance (Bornstein et al., 2014; Lee, 2011; Rowe, 2012). We also observed that at age 2–4 years, children's lexical diversity correlated with their cognitive ability, and the latter, in turn, predicted children's academic performance four years later. This pattern of associations suggests overall that cognitive, language and academic abilities comprise interrelated aspects of the same developmental domain (Peng & Kievit, 2020; Peng et al., 2019). By contrast, we observed no stability in children's behavioural measures across time and assessment methods in the current study. This finding suggests that children's differences in behavioural tendencies may be more difficult to reliably capture with psychological measures than their cognitive traits.

Second, our results differ from previous findings that early life language experiences are associated with children's language development (Gilkerson et al., 2018; Hart & Risley, 1995; Marchman & Fernald, 2008), although they are not exceptional (Merz et al., 2020; Wang et al., 2020). We speculate that methodological and sampling differences potentially account for much of the observed discrepancy in the results. We discuss three of these differences in detail here. For one, empirical studies in this area employed different observation methods and observation periods, with some extracting data from video‐taped interactions (Vallotton et al., 2017), others from parent reports of child language (Dale et al., 2020) and again others from audio‐recordings of typical family life (Gilkerson et al., 2018; Griffin & Morrison, 1997; Marchman & Fernald, 2008). As a result, the studies vary considerably in their statistical power to detect meaningful associations that replicate across observation methods (see d'Apice et al. (2019) for a review). For the other, samples have differed in their socio‐demographic characteristics across studies. For example, the current sample was fairly homogenous in terms of marital status, education levels and socio‐economic status (SES), which possibly resulted in reduced variability and thus, associations that may have been meaningful elsewhere could not be detected here. Other studies in this area analysed data from samples of lower SES (Lee, 2011; Lonigan, rgss, & Anthony, 2000) and with more varied educational backgrounds (Gilkerson et al., 2018; Merz et al., 2020). That said, Merz et al., (2020) also found no associations between children's language experiences and later reading ability, similar to the findings of the present study, despite testing a sample that was diverse in SES and education. Finally, it is possible that we failed to detect an association of early life parenting and language experiences with child development outcomes, because they only become salient during specific developmental periods. Several previous studies observed age‐specific links between early life environments and children's performance in developmental tasks (Broberg et al., 1997; Griffin & Morrison, 1997; Melhuish et al., 2008; Rowe, 2012; Vallotton et al., 2017). It is possible that developmental periods moderate the effect of parenting and language exposures onto child developmental outcomes.

Third, we observed an association between positive parenting and children's behavioural difficulties, as well as contemporaneous associations (i.e., at T1) between early life parenting and children's internalizing and externalizing behaviour problems. These findings confirm that parenting is associated with children's behavioural development (Akcinar & Baydar, 2016; Boeldt et al., 2012; Denham et al., 2000), although the direction of association was unexpected. We found that positive parenting led to greater behavioural difficulties rather than serving as a buffer. Our study design only allows speculating about the explanation for this finding; future research must explore, for example, if children with behaviour problems evoke more positive parenting (i.e., responsiveness of parents). Furthermore, we observed no associations between early life parenting and children's later behaviour academic performance, inconsistent with some previous findings (Chen et al., 1997; Grolnick & Ryan, 1989) but in line with others (Gunderson et al., 2018; NICHD Early Child Care Research Network, 2004). Again, future research will be essential to better elucidate the relation between parenting and academic performance in middle childhood.

Finally, we observed comparatively strong links between attitudes towards school and behavioural difficulties and prosocial behaviour. Children in the present study with greater behavioural difficulties also had more negative attitudes to school, while children with more prosocial behaviours showed positive attitudes to school. We note that, by contrast, behavioural difficulties and prosocial behaviour were not associated with actual school performance. It is plausible that children's behavioural adjustment affects their attitudes towards school (Huang & Anyon, 2020), which may be one possible pathway through which behaviours affect academic performance. Attitudes towards school and learning are malleable (Blackwell et al., 2007) and thus, may constitute good targets for interventions that seek to improve children's school performance, especially when they suffer from behavioural adjustment problems.

### Strengths and limitations

The current study's strengths include its naturalistic observation data that spanned three days and its longitudinal design. However, it is not without limitations. First, some of our measures could have had better validity, for example children's academic achievement could have been reported by teachers or extracted from the children's official performance records. Also, some of our variables were not normally distributed with ceiling and flooring effects, although the majority of variables met the assumption of normality. Second, we used different assessment methods across T1 and T2; for example, researchers rated children's behaviour on the basis of naturalistic audio‐recordings at T1, but at T2 behavioural difficulties and prosocial behaviour were assessed through parent reports. Using different methods across assessment waves may have obliterated some of the expected associations. Also, our data would have been stronger had it been possible to collect data directly from children at T2, like we did at T1. Third, the children in our sample ranged in age by up to 19 months and T1 and up to 33 months at T2. Although similar age ranges are not uncommon in other studies in this area (Christakis et al., 2009; Gilkerson et al., 2018; Lonigan et al., 2000) and we adjusted our analyses for children's age differences at T1 and T2, they may still cause residual confounding. Future studies should test samples that are more consistent in age. Fourth, we assessed children's language experiences at T1 in terms of the number and lexical diversity of adult words that they were exposed to, rather than focusing on child‐directed speech, which some have argued is key for children's language development (Hart & Risley, 1995; Rowe, 2008). Likewise, we assessed parenting at T1 via observer ratings of excerpts of audio‐recordings that cannot capture some important parenting behaviours, such as facial expressions, touch and other nonverbal means. Thus, we may not have observed the expected associations because we assessed not all early life experiences that are relevant to children's development. Finally, our sample was fairly homogenous with regards to families' socio‐demographic characteristics. Because our sample was on average of higher SES and education than the wider population of Britain, generalizing our findings to children and families of lower SES is difficult.

## CONCLUSIONS

In naturalistic observation data collected from British families, we found no association between children's early life experiences of language and parenting and their cognitive ability, academic performance and behavioural outcomes during middle childhood. Naturalistic data tend to be ‘noisier’ than lab‐based observations and measures collected through adult surveys, in the way that they capture a multitude of factors, some of which are meaningful and some of which only cause measurement error. This condition does not, however, invalidate naturalistic research approaches (Purpura, 2019) but urges developmental scientists to investigate why results vary across methods to achieve a more complete picture of children's developmental differences. Future research that seeks to explore the role of early life experiences for development should employ multiple observation methods that allow collecting precise, in‐depth naturalistic data from large, representative samples that afford good statistical power. The data that emerge from such research are pivotal for establishing an empirical evidence‐base that permits drawing conclusions about the effects of early life experiences on child development.

## AUTHOR CONTRIBUTIONS

**Sophie von Stumm:** Conceptualization; formal analysis; funding acquisition; methodology; project administration; supervision; writing – original draft; writing – review and editing. **Jelena O'Reilly:** Writing – original draft; writing – review and editing. **katrina d'apice:** Writing – review and editing.

## Funding information

SvS is recipient of a CRISP Jacobs Fellowship and a British Academy Mid‐Career Fellowship. This work was also supported by the University of York.

## CONFLICT OF INTEREST

Author declare no conflict of interest.

### OPEN RESEARCH BADGES

This article has earned a Preregistered Research Designs badge for having a preregistered research design, available at https://osf.io/y9tn4/

## Supporting information


**Table S1.** Multiple Linear Regression Predicting T2 Language from T1 Measures and Controls.
**Table S2.** Multiple Linear Regression Predicting T2 Language from T1 and T2 Measures and Controls.
**Table S3.** Multiple Linear Regression Predicting T2 Academic Performance from T1 Measures and Controls.
**Table S4.** Multiple Linear Regression Predicting T2 Academic Performance from T1and T2 Measures and Controls.
**Table S5.** Multiple Linear Regression Predicting T2 School Attitude from T1 Measures and Controls.
**Table S6.** Multiple Linear Regression Predicting T2 School Attitude from T1 and T2 Measures and Controls.
**Table S7.** Multiple Linear Regression Predicting T2 Total Difficulties Score from T1 Measures and Controls.
**Table S8.** Multiple Linear Regression Predicting T2 Total Difficulties Score from T1 and T2 Measures and Controls.
**Table S9.** Multiple Linear Regression Predicting T2 Prosocial Behavior from T1 Measures and Controls.
**Table S10.** Multiple Linear Regression Predicting T2 Prosocial Behavior from T1 and T2 Measures and Controls.

## Data Availability

Data are not publicly available because of their sensitive nature (i.e., day‐long naturalistic home observations). This study was preregistered: https://osf.io/y9tn4/
