# Supplementary material for: Predicting developmental outcomes in middle childhood from early life language and parenting experiences
Source: Br J Dev Psychol. 2022 Jul 26;40(4):487–503. doi: 10.1111/bjdp.12427 (PMC9796835; doi:10.1111/bjdp.12427)
Supplement: Supplementary file 1 — Table S1. Multiple Linear Regression Predicting T2 Language from T1 Measures and Controls. Table S2. Multiple Linear Regression Predicting T2 Language from T1 and T2 Measures and Controls. Table S3. Multiple Linear Regression Predicting T2 Academic Performance from T1 Measures and Controls. Table S4. Multiple Linear Regression Predicting T2 Academic Performance from T1and T2 Measures and Controls. Table S5. Multiple Linear Regression Predicting T2 School Attitude from T1 Measures and Controls. Table S6. Multiple Linear Regression Predicting T2 School Attitude from T1 and T2 Measures and Controls. Table S7. Multiple Linear Regression Predicting T2 Total Difficulties Score from T1 Measures and Controls. Table S8. Multiple Linear Regression Predicting T2 Total Difficulties Score from T1 and T2 Measures and Controls. Table S9. Multiple Linear Regression Predicting T2 Prosocial Behavior from T1 Measures and Controls. Table S10. Multiple Linear Regression Predicting T2 Prosocial Behavior from T1 and T2 Measures and Controls. [file BJDP-40-487-s001.docx]

**Supporting information for: Predicting developmental outcomes in middle childhood from early life language and parenting experiences**

**Table S1**

*Multiple Linear Regression Predicting T2 Language from T1 Measures and Controls*

| Model 1 (DVAP) | | | | | |
| --- | --- | --- | --- | --- | --- |
|  | Estimate | *Std. E* | *t* | *p* | Beta |
| (Intercept) | 42.56 | 17.78 | 2.39 | .019 |  |
| Birth Order | 1.15 | 1.22 | 0.93 | .352 | .12 |
| SES index | 0.14 | 1.28 | 0.11 | .913 | .01 |
| Adult word counts | 0.00 | 0.00 | 0.76 | .450 | .09 |
| Adult lexical diversity | 0.04 | 0.06 | 0.71 | .482 | .08 |
| Positive parenting | 0.71 | 1.48 | 0.48 | .633 | .06 |
| Critical parenting | 3.88 | 14.83 | 0.26 | .794 | .03 |
| *R*^2^ adj. | -.05 |  |  |  |  |
| *F* | 0.35 (*df* = 6, 79) | |  |  |  |

**Table S2**

*Multiple Linear Regression Predicting T2 Language from T1 and T2 Measures and Controls*

| Model 2 (DVAP) | | | | | |
| --- | --- | --- | --- | --- | --- |
|  | Estimate | *Std. E* | *t* | *p* | Beta |
| (Intercept) | 73.66 | 51.44 | 1.43 | .156 |  |
| Birth Order | 1.57 | 1.27 | 1.24 | .221 | .16 |
| SES index | -0.28 | 1.44 | -0.19 | .846 | -.02 |
| Adult word counts | 0.00 | 0.00 | 0.54 | .589 | .07 |
| Adult lexical diversity | 0.01 | 0.07 | 0.16 | .874 | .02 |
| Positive parenting | -0.17 | 1.55 | -0.11 | .912 | -.01 |
| Critical parenting | 3.64 | 15.66 | 0.23 | .817 | .03 |
| Child lexical diversity | 0.08 | 0.045 | 1.86 | .067 | .25 |
| PARCA | 0.96 | 0.77 | 1.25 | .215 | .16 |
| Internalizing behavior | 6.37 | 6.22 | 1.02 | .309 | .12 |
| Externalizing behavior | -12.21 | 9.37 | -1.30 | .197 | .17 |
| *R*^2^ adj. | .01 |  |  |  |  |
| *F* | 1.06 (*df* = 10; 72) | |  |  |  |

**Table S3**

*Multiple Linear Regression Predicting T2 Academic Performance from T1 Measures and Controls*

| Model 1 (Academic Performance) | | | | | |
| --- | --- | --- | --- | --- | --- |
|  | Estimate | *Std. E* | *t* | *p* | Beta |
| (Intercept) | 57.17 | 47.88 | 1.19 | .236 |  |
| Birth Order | 1.01 | 3.32 | 0.30 | .763 | .04 |
| SES index | -1.13 | 3.69 | -0.31 | .761 | -.04 |
| Adult word counts | 0.00 | 0.00 | 0.51 | .612 | .06 |
| Adult lexical diversity | -0.06 | 0.17 | -0.38 | .708 | -.05 |
| Positive parenting | -2.97 | 3.98 | -0.75 | .457 | -.10 |
| Critical parenting | 29.56 | 40.06 | 0.74 | .463 | .09 |
| *R*^2^ adj. | -.06 |  |  |  |  |
| *F* | 0.29 (*df* = 6; 75) | |  |  |  |

**Table S4**

*Multiple Linear Regression Predicting T2 Academic Performance from T1and T2 Measures and Controls*

| Model 2 (Academic Performance) | | | | | |
| --- | --- | --- | --- | --- | --- |
|  | Estimate | *Std. E* | *t* | *p* | Beta |
| (Intercept) | 236.31 | 136.07 | 1.74 | .087 |  |
| Birth Order | -0.01 | 3.19 | -0.00 | .999 | -.00 |
| SES index | -0.53 | 3.74 | -0.14 | .887 | -.07 |
| Adult word counts | -0.00 | 0.00 | -0.54 | .589 | -.07 |
| Adult lexical diversity | 0.01 | 0.17 | 0.09 | .932 | .01 |
| Positive parenting | -6.10 | 3.90 | -1.56 | .124 | -.19 |
| Critical parenting | 10.11 | 39.14 | 0.26 | .797 | .03 |
| Child lexical diversity | -0.03 | 0.12 | -0.23 | .822 | -.03 |
| PARCA | 7.98 | 2.01 | 3.97 | .000 | .51 |
| Internalizing behavior | 6.86 | 15.84 | 0.43 | .667 | .05 |
| Externalizing behavior | -36.06 | 24.27 | -1.49 | .142 | -.19 |
| *R*^2^ adj. | .10 |  |  |  |  |
| *F* | 1.91 (*df* = 10; 69) | |  |  |  |

**Table S5**

*Multiple Linear Regression Predicting T2 School Attitude from T1 Measures and Controls*

| Model 1 (School Attitude) | | | | | |
| --- | --- | --- | --- | --- | --- |
|  | Estimate | *Std. E* | *t* | *p* | Beta |
| (Intercept) | 15.84 | 5.14 | 3.11 | .003 |  |
| Birth Order | 0.29 | 0.35 | 0.81 | .420 | .10 |
| SES index | -0.09 | 0.41 | -0.24 | .811 | -.03 |
| Adult word counts | 0.00 | 0.00 | -0.24 | .820 | -.03 |
| Adult lexical diversity | -0.03 | 0.02 | -1.41 | .164 | -.17 |
| Positive parenting | -0.51 | 0.42 | -1.12 | .271 | -.14 |
| Critical parenting | -2.03 | 4.32 | -0.47 | .640 | -.06 |
| *R*^2^ adj. | -.02 |  |  |  |  |
| *F* | 0.73 (*df* = 6; 73) | |  |  |  |

**Table S6**

*Multiple Linear Regression Predicting T2 School Attitude from T1 and T2 Measures and Controls*

| Model 2 (School Attitude) | | | | | |
| --- | --- | --- | --- | --- | --- |
|  | Estimate | *Std. E* | *t* | *p* | Beta |
| (Intercept) | 22.99 | 15.43 | 1.49 | .141 |  |
| Birth Order | 0.26 | 0.36 | 0.72 | .472 | .09 |
| SES index | -0.104 | 0.43 | -0.24 | .810 | -.03 |
| Adult word counts | 0.00 | 0.00 | -0.67 | .507 | -.09 |
| Adult lexical diversity | -0.02 | 0.02 | -1.03 | .309 | -.14 |
| Positive parenting | -0.73 | 0.44 | -1.64 | .105 | -.23 |
| Critical parenting | -4.43 | 4.52 | -0.98 | .331 | -.13 |
| Child lexical diversity | 0.00 | 0.014 | 0.30 | .762 | .04 |
| PARCA | 0.58 | 0.23 | 2.18 | .033 | .31 |
| Internalizing behavior | 2.25 | 1.79 | 1.26 | .214 | .16 |
| Externalizing behavior | -3.07 | 2.75 | -1.12 | .268 | -.15 |
| *R*^2^ adj. | .01 |  |  |  |  |
| *F* | 1.07 (*df* = 10; 67) | |  |  |  |

**Table S7**

*Multiple Linear Regression Predicting T2 Total Difficulties Score from T1 Measures and Controls*

| Model 1 (Total Difficulties Score) | | | | | |
| --- | --- | --- | --- | --- | --- |
|  | Estimate | *Std. E* | *t* | *p* | Beta |
| (Intercept) | -8.53 | 11.05 | -0.77 | .440 |  |
| Birth Order | -1.25 | 0.76 | -1.65 | .104 | -.19 |
| SES index | -0.25 | 0.79 | -0.32 | .754 | -.03 |
| Adult word counts | 0.00 | 0.00 | 0.07 | .947 | .01 |
| Adult lexical diversity | 0.06 | 0.04 | 1.49 | .141 | .17 |
| Positive parenting | 1.99 | 0.92 | 2.04 | .044 | .24 |
| Critical parenting | 5.64 | 9.22 | 0.61 | .542 | .07 |
| *R*^2^ adj. | .05 |  |  |  |  |
| *F* | 1.82 (*df* = 6; 79) | |  |  |  |

**Table S8**

*Multiple Linear Regression Predicting T2 Total Difficulties Score from T1 and T2 Measures and Controls*

| Model 2 (Total Difficulties Score) | | | | | |
| --- | --- | --- | --- | --- | --- |
|  | Estimate | *Std. E* | *t* | *p* | Beta |
| (Intercept) | -38.51 | 32.19 | -1.19 | .236 |  |
| Birth Order | -0.97 | 0.86 | -1.21 | .223 | -.15 |
| SES index | -0.83 | 0.90 | -0.92 | .362 | -.11 |
| Adult word counts | 0.00 | 0.00 | -0.24 | .811 | -.03 |
| Adult lexical diversity | 0.02 | 0.04 | 0.41 | .621 | .06 |
| Positive parenting | 2.38 | 0.12 | 2.45 | .017 | .31 |
| Critical parenting | 6.55 | 9.78 | 0.66 | .506 | .08 |
| Child lexical diversity | 0.03 | 0.03 | 0.94 | .352 | .12 |
| PARCA | -0.20 | 0.91 | -0.43 | .672 | -.05 |
| Internalizing behavior | -3.59 | 3.91 | -0.92 | .351 | -.11 |
| Externalizing behavior | 9.41 | 5.86 | 1.60 | .113 | .29 |
| *R*^2^ adj. | .05 |  |  |  |  |
| *F* | 1.45 (*df* = 10; 72) | |  |  |  |

**Table S9**

*Multiple Linear Regression Predicting T2 Prosocial Behavior from T1 Measures and Controls*

| Model 1 (Prosocial Behavior) | | | | | |
| --- | --- | --- | --- | --- | --- |
|  | Estimate | *Std. E* | *t* | *p* | Beta |
| (Intercept) | 7.84 | 5.02 | 1.56 | .122 |  |
| Birth Order | -0.69 | 0.35 | -2.00 | .049 | -.24 |
| SES index | 0.58 | 0.36 | 1.59 | .115 | .19 |
| Adult word counts | 0.00 | 0.00 | 0.00 | .997 | .00 |
| Adult lexical diversity | -0.02 | 0.02 | -1.41 | .163 | -.16 |
| Positive parenting | -0.32 | 0.42 | -0.77 | .447 | -.11 |
| Critical parenting | 4.84 | 4.25 | 1.16 | .251 | .13 |
| *R*^2^ adj. | .03 |  |  |  |  |
| *F* | 1.44 (*df* = 6; 79) | |  |  |  |

**Table S10**

*Multiple Linear Regression Predicting T2 Prosocial Behavior from T1 and T2 Measures and Controls*

| Model 2 (Prosocial Behavior) | | | | | |
| --- | --- | --- | --- | --- | --- |
|  | Estimate | *Std. E* | *t* | *p* | Beta |
| (Intercept) | 7.37 | 14.68 | 0.51 | .615 |  |
| Birth Order | -0.77 | 0.36 | -2.13 | .037 | -.27 |
| SES index | 0.76 | 0.41 | 1.94 | .068 | .22 |
| Adult word counts | 0.00 | 0.00 | 0.37 | .716 | .05 |
| Adult lexical diversity | -0.02 | 0.02 | -0.82 | .414 | -.10 |
| Positive parenting | -0.45 | 0.44 | -1.03 | .307 | -.13 |
| Critical parenting | 3.75 | 4.44 | 0.84 | .401 | .10 |
| Child lexical diversity | 0.00 | 0.01 | 0.10 | .921 | .01 |
| PARCA | 0.09 | 0.22 | 0.42 | .676 | .05 |
| Internalizing behavior | 2.82 | 1.85 | 1.56 | .123 | .18 |
| Externalizing behavior | -2.54 | 2.66 | -0.96 | .342 | -.12 |
| *R*^2^ adj. | .04 |  |  |  |  |
| *F* | 1.33 (*df* = 10; 72) | |  |  |  |
